# Manipulation of Lateral Pharyngeal Wall Muscles in Sleep Surgery: A Review of the Literature

**DOI:** 10.3390/ijerph17155315

**Published:** 2020-07-23

**Authors:** Giovanni Cammaroto, Luigi Marco Stringa, Giannicola Iannella, Giuseppe Meccariello, Henry Zhang, Ahmed Yassin Bahgat, Christian Calvo-Henriquez, Carlos Chiesa-Estomba, Jerome R. Lechien, Maria Rosaria Barillari, Bruno Galletti, Francesco Galletti, Francesco Freni, Cosimo Galletti, Claudio Vicini

**Affiliations:** 1 Head and Neck Department, ENT & Oral Surgery Unit, G.B. Morgagni, L. Pierantoni Hospital, Forlì, FC 47100 ASL of Romagna, Italy; giannicolaiannella@hotmail.it (G.I.); giuseppemec@yahoo.it (G.M.); claudio@claudiovicini.com (C.V.); 2 Young Otolaryngologists-International Federations of Oto-rhinolaryngological Societies (YO-IFOS), 75000 Paris, France; christian.calvo.henriquez@gmail.com (C.C.-H.); chiesaestomba86@gmail.com (C.C.-E.); Jerome.LECHIEN@umons.ac.be (J.R.L.); mariarosaria.barillari@unicampania.it (M.R.B.); 3 Department of Otolaryngology, Head and Neck Surgery, University of Ferrara, FE 44121 Ferrara, Italy; luigi.stringa@gmail.com; 4 Department of Otolaryngology, Head and Neck, Royal London Hospital, London E1 1FR, UK; henryzhang87@gmail.com; 5 Department of Otorhinolaryngology, Alexandria University, Alexandria 21526, Egypt; ahmedyassinbahgat@gmail.com; 6 Department of otolaryngology, University Hospital Complex of Santiago de Compostela, 15706 Santiago de Compostela, Spain; 7 Department of Otorhinolaryngology, Head & Neck Surgery, Hospital Universitario Donostia, 20014 San Sebastian, Spain; 8 Department of Otolaryngology, Head & Neck Surgery, Foch Hospital, School of Medicine, UFR Simone Veil, Université Versailles Saint-Quentin-en-Yvelines (Paris Saclay University), 75000 Paris, France; 9 Department of Mental and Physical Health and Preventive Medicine, University of L. Vanvitelli, CE 81100 Naples, Italy; 10 Department of Adult and Development Age Human Pathology “Gaetano Barresi”, Unit of Otorhinolaryngology, University of Messina, ME 98125 Messina, Italy; bgalletti@unime.it (B.G.); fgalletti@unime.it (F.G.); ffreni@unime.it (F.F.); 11 Comprehensive Dentistry Department, Faculty of Dentistry, Universitat de Barcelona, L’Hospitalet de Llobregat (Barcelona), 08907 Catalonia, Spain; cosimo_galletti@hotmail.it; 12 ENT department, University of Ferrara, FE 44121 Ferrara, Italy

**Keywords:** OSA, pharyngoplasty, sleep surgery, pharynx

## Abstract

Background: Obstructive sleep apnea syndrome (OSAS) occurs due to upper airway obstruction resulting from anatomical and functional abnormalities. Upper airway collapsibility, particularly those involving the lateral pharyngeal wall (LPW), is known to be one of the main factors contributing to the pathogenesis of OSAS, leading the authors of the present study to propose different strategies in order to stiffen the pharyngeal walls to try to restore normal airflow. Methods: An exhaustive review of the English literature on lateral pharyngeal wall surgery for the treatment of OSAS was performed using the PubMed electronic database. Results: The research was performed in April 2020 and yielded approximately 2000 articles. However, considering the inclusion criteria, only 17 studies were included in the present study. Conclusions: The analyzed surgical techniques propose different parts of LPW on which to focus and a variable degree of invasivity. Despite the very promising results, no gold standard for the treatment of pharyngeal wall collapsibility has been proposed. However, thanks to progressive technological innovations and increasingly precise data analysis, the role of LPW surgery seems to be crucial in the treatment of OSAS patients.

## 1. Introduction

Obstructive sleep apnea syndrome (OSAS) is a very common health problem characterized by absent or insufficient ventilation during sleep as a consequence of the multilevel structural collapse of the upper airways, which usually involves the velopharynx, base of the tongue, and lateral pharyngeal walls. The diagnosis of OSAS is the result of the integration of anamnestic and clinical evaluations with instrumental data provided by polysomnography (PSG), which, collecting physiological parameters during sleep, allows a deeper and objective assessment of sleep-related breathing disorders. In order to localize the obstacle compromising correct airflow, a specific examination (like Müller’s maneuver) can be performed when undergoing fiberoptic laryngoscopy and drug-induced sleep endoscopy (DISE). Described by different authors as a key point in the determination of airflow, the lateral pharyngeal wall (LPW) has been demonstrated to present an increased thickness and collapsibility in patients affected by OSAS, resulting in a potential cause of airway obstruction [1,2,3,4]. LPW includes muscular structures such as the palatoglossus muscle (PGM), the palatopharyngeal muscle (PPM), the superior pharyngeal constrictor (SPC), and, ultimately, lymphatic tissue, the palatine tonsils. Stabilizing LPW might be achieved by means of mandibular advancement devices or surgery [5]. Different authors have proposed surgical solutions to prevent the upper airways from collapsing [6], but in 2003, Cahali [7], for the first time, described lateral pharyngoplasty (LP), i.e., a surgical transoral approach focused on LPW, allowing, in this way, an enlarging of the oropharyngeal space and a stiffening of fibromuscular structures. Following on, surgeons have developed several techniques targeting the modification of LPW, trying to reduce surgical invasivity and complications, but none of them have been indicated as a gold standard procedure by the scientific community so far.

The efficacy of palate surgery is well documented by a meta-analysis published in 2018 by Pang et al. [8]. However, no reviews that are mainly oriented on lateral pharyngoplasty have been published so far. The aim of our study is to present a systematic review of the literature regarding the surgical techniques involving modifications of LPW for the treatment of OSAS in order to show their surgical stages, technical aspects, as well as their advantages and inconveniences, to better understand their applications on and contributions to the correction of upper airway obstruction.

## 2. Materials and Methods 

A thoughtful review of the English language literature on the surgical procedures targeting LPW for the management of sleep apnea was performed using PubMed, EMBASE, Cochrane, and CENTRAL electronic databases. Two searches using (1) pharyngoplasty sleep apnea and (2) palate surgery sleep apnea as keyword clusters were performed, and they were combined with the use of the AND function to better select the research. Each paper included in the study met the following inclusion criteria: (1) the surgical treatment of OSAS patients, (2) the application of surgical procedures designed for structural modification of LPW, (3) the presence of instrumental parameters acquired by PSG during preoperative and postoperative evaluations, (4) reports including patients previously treated with Continuous Positive Airway Pressure (CPAP), and (5) reports including patients who did not undergo previous sleep surgery procedures. Articles that were not in accordance with the inclusion criteria were excluded. The subsequent criteria were applied with the aim of excluding inappropriate studies: surgical technique reports without significant outcome data, papers consisting of meta-analyses or literature reviews on surgical procedures, studies relative to ablative techniques, and articles describing animal or cadaveric samples. In order to further reduce the risk of incomplete literature analysis, a manual search through the bibliography of the included papers was carried out.

With the purpose of obtaining an organized and exhaustive review, the Preferred Reporting Items for Systematic Reviews and Meta-Analyses (PRISMA) criteria were applied first to select the papers and, secondly, to stratify them according to their level of evidence [9].

## 3. Results

**Figure 1:**
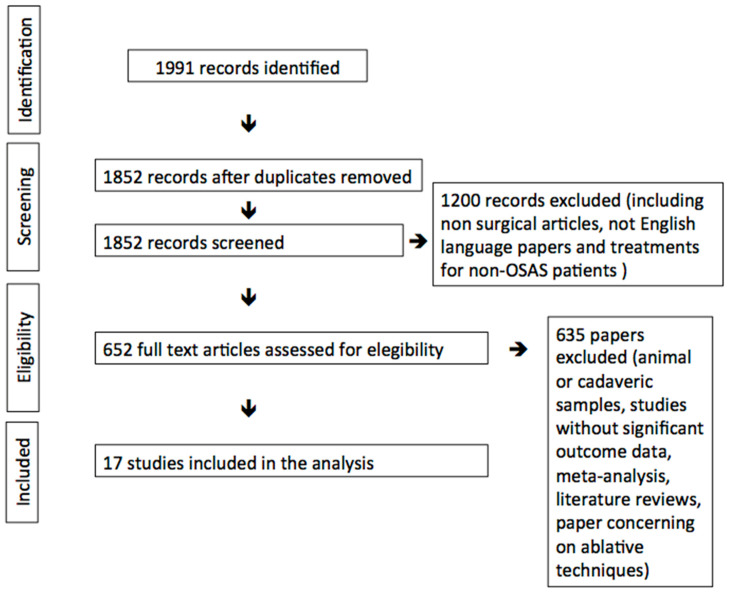
Preferred Reporting Items for Systematic Reviews and Meta-Analyses (PRISMA).

In April 2020, the research led to the detection of approximately 2000 articles (from 1980 to 2020), but in accordance with the inclusion and exclusion criteria, 17 articles were enrolled in the present review. As the articles show detailed descriptions of surgical procedures to better clarify their singularities and effects, we grouped each article in accordance to which component of LPW is mostly involved and how deeply its modification occurs. As a result, we found four articles presenting surgical procedures in which SPC is manipulated and/or dissected, while in the remaining papers, PPM is the main structural target. In particular, five studies proposed a total or subtotal section of the muscle, four papers a partial section of the muscular fibers, and in four studies, minimal tissue handling is performed (Table 1, Figure 1). 

Data on the efficacy and complications of LPW surgeries are shown in Table 1. The majority of samples included in the study consisted of patients affected by severe OSAS (mean pre-op apnea–hypopnea index (AHI) > 30). A mean reduction of more than 50% of AHI and a mean post-op AHI < 20 were observed in all series except one [10]. Some authors did not specify complications and their rates.

## 4. Discussion

As suggested by the literature, some OSAS patients present structural and functional abnormalities in the upper airways. In these patients, the pharynx (specifically, LPW) is thicker and more collapsible upon exposure to negative pressure during inspiration [4,5]. In 1981, Fujita et al. [6] introduced, for the first time, the uvulopalatopharyngoplasty (UPPP)—a surgical procedure finalized at the enlargement of the pharyngeal space—but the association of postoperative complications and dissatisfactory results drove surgeons to alternative approaches. Thus, LPW acquired a central role in OSAS pathogenesis, drawing the attention of numerous studies. Several surgical techniques have been proposed to enlarge and stiffen the pharyngeal tract. All of the procedures are performed under general anesthesia, with either nasotracheal or orotracheal intubation, while the patient is placed in the supine position and a mouth gag is applied to expose the oropharynx. What differentiates these techniques is the degree of involvement of the different LPW components, and how radical their modifications are.

Taking into account the sample sizes and the average postoperative follow up of the included articles, the following findings need to be highlighted.

Our review shows that the majority of the evaluated articles included patients with severe OSAS who experienced a significant postoperative reduction of AHI. These data show that the severity of sleep apneas should not be considered an exclusion criterion for LPW surgery.

However, the selection process of surgical candidates in each study was partially investigated in this review. The choice of performing preoperative DISE might influence the outcomes, and therefore, more attention should be dedicated to this aspect in future studies.

In our opinion, the main strength of our review is its specific focus on the muscular structures that are manipulated in each surgical technique. A better understanding of the involvement of the LPW muscles and prospective comparative studies might allow sleep surgeons to select the most effective surgical procedure. 

### 4.1. Superior Pharyngeal Constrictor 

In 2003, Cahali [7] presented LP as the first surgical procedure targeted to modify LPW for the treatment of pharyngeal instability, and SPC was the principal muscular structure involved. More precisely, after the individuation of SPC by bilateral tonsillectomy or mucosal removal of tonsillar fossa in the case of a previous tonsillectomy, it is split by a craniocaudal section into two flaps, one medially based, and the other, laterally based. The latter is sutured anteriorly to PGM. In order to obtain more space and simultaneously reduce traction forces, a palatal incision is performed from the lateral base of the uvula, extending laterally and superiorly, leading to the isolation of the upper part of PPM, which is partially sectioned in this portion. After that, two flaps are obtained: one superior flap, which is sutured in a Z-plasty fashion with the palatal flap, and one inferior flap, which is sutured to the anterior tonsillar pillar. The same procedure is performed on both sides. Although this innovative technique has shown satisfactory results in the correction of upper airway obstruction, it has been described to be related to transient oronasal reflux and partial taste loss. Despite the possible swallowing complication presented by LP, other authors consider SPC a successful target. José Antonio Pinto et al. [10], in so-called lateral-expansion pharyngoplasty, suggested the association of LP with expansion sphincter pharyngoplasty, presented for the first time by Pang and Woodson. In this way, the SPC section is combined with the section of PP muscle and the further suture of its cranial flap to the hamulus of the pterygoid process in order to improve the retropalatal obstruction. In their series of 38 samples, no patient suffered major complications. Regarding the modification of SPC, not all authors have pursued its section, and in some cases, a more conservative approach has been suggested. Huseh-Yu Li and Li-Ang Lee [11] introduced relocation pharyngoplasty, in which, once identified, SPC is grasped and sutured to PGM. At the same time, an elliptical cut from the lateral base of the uvula, extending superolaterally, is performed and the mucosal and submucosal adipose tissue is removed in order to gain further space, reducing tension forces. Once PPM is isolated from SPC, the posterior pillar is sewn to PGM. A distal mucosal resection of the uvula is also associated. Likewise, in anterolateral advancement pharyngoplasty by Emara et al., a relocation of SPC is performed without any muscular fiber section [12]. In particular, a limited dissection and partial separation of anterior and posterior parts of PPM from SPC were performed in the upper part of the tonsillar fossa. Then, SPC was plicated with a mattress-style suture and together with PPM (just inferiorly to the confluence point of its anterior and posterior parts) are anchored to the pterygomandibular raphe. Finally, the upper half of the posterior part of PPM is sutured to the levator veli palatini muscle. 

### 4.2. Palatopharyngeal Muscle

After the very promising results showed by LP, many authors started to elaborate on new approaches aimed at the same outcomes, trying to avoid the complications. The manipulations of SPC were attributed as a significant source of postoperative swallowing problems, and in this way, PPM was considered a proper target in order to strengthen LPW. As shown below, a complete muscular fiber section is not performed in all the procedures, and a variable degree of tissue sparing is applied.

#### 4.2.1. Total/Subtotal Section of Muscular Fibers

Three years after the introduction of LP, devising a surgical procedure that is easy to perform with a low rate of complications, K. Pang and T. Woodson [13] introduce the so-called expansion sphincter pharyngoplasty (ESP). In this new approach, all patients are submitted to bilateral tonsillectomy in order to identify PPM. Then, its muscular fibers are sectioned at the inferior end, and the posterior surface of the superior flap is partially detached from SPC. In order to isolate the soft palate muscles, a superolateral incision is performed on the anterior pillar. PPM is then lifted superolaterally, suturing it to the soft palate muscles. A partial uvulectomy is then performed. This technique showed very encouraging results and traced the path to more innovative approaches. A conservative modification of ESP was presented in 2013 by G. Sorrenti and O. Piccin, named functional expansion pharyngoplasty (FEP) [14]. The most important variation applied to the original technique is the replacement of the superolateral incision of soft palatal mucosa to expose the palatal muscles with a preparation of a tunnel through the palatal musculature from the apex to the hamulus of the tonsillar fossa of the pterygoid process. Once sectioned and rotated, the PPM flap is elevated through the tunnel and anchored to the palatine muscles close to the hamulus. A thin rim of muscular fibers of PPM is preserved medially to avoid retracting scar tissue at the posterior pillar. In this way, a more physiological widening force is applied to LPW, reducing tissue dissection and the subsequent retracting scar. Some years later, Sorrenti et al. [25] added some technical updates to their technique. In particular, they proposed the use of knotless barbed V-Loc sutures to fix the PPM flap to the apex of the mandibular pterygoid fold. Compared to previous FEP, this technique became easier and faster to perform with a powerful docking site. In order to reduce tissue manipulation and postoperative complications linked to tonsillectomy, A.M.M.E. Albassiouny, in 2014 [15], introduced the so-called soft palatal posterior pillar webbing flap palatopharyngoplasty technique in which the palatine tonsils are spared, and a transverse section of PPM is performed. Specifically, once the ventral mucosa of the posterior pillar is removed, it is sectioned in two points—one lateral, including PPM, and one medial, close to the uvula—in order to obtain two flaps. The lateral flap is then sutured to the most superolateral part of the anterior pillar, lateralizing the tonsil. After a submucosal dissection of the palatal mucosa and a shortening of the medial flap in length, it is turned up and sutured to the free margin of the soft palate. Two years later, A.M.M.E. Albassiouny [16] proposed two modifications to his original approach: coblation-assisted extracapsular tonsillectomy when tonsil collapse is documented by DISE, and the use of barbed STRATAFIX sutures in order to better control the tension impressed on the pharyngeal structure. In both cases, postoperative temporary velopharyngeal insufficiency and excessive postnasal discharge have been reported.

#### 4.2.2. Partial Section of Muscular Fibers

The progressive interest in the treatment of OSAS has led to the elaboration of an increasingly conservative approach, allowing its application to a more delicate population such as pediatrics. On the heels of the excellent outcomes shown first by K. Pang and T. Woodson, and later by G. Sorrenti and O. Piccin, S.O. Ulualp [17] presented a modified ESP, applied to a pediatric population. In fact, in order to reduce tissue removal, after bilateral tonsillectomy, a partial section of the PPM anterior fibers is performed at the junction of the upper third and mid-third. Then, superolateral tunneling of the soft palate is prepared, and the upper portion of PPM is pulled up in the muscular tunnel and sutured to it. Not only the lateral side but also the anterior portion of the soft palate has been reported to be modified during LPW surgery. In this regard, M.J. Kim et al. [18] introduced a modified uvulopalatal flap with a partial LP in order to widen the retropalatal space anteroposteriorly and transversely. The posterior pillar is cut at its junction with the uvula, and once trimmed, it is sutured to the anterior pillar. After having calculated the mucosa and submucosal fat to be removed by measuring the halfway point between the junction of the soft and hard palates and the tip of the uvula, a diamond-shaped area of the soft palate is removed. Then, a suture of the muscular and mucosal rims is performed. In some cases, a bilateral tonsillectomy is performed, and the patients are submitted to concomitant nasal septoplasty and turbinate surgery. Following the interest in conservative approaches, and thanks to material technology improvements, in 2015, C. Vicini et al. [19] introduced an innovative tissue-sparing approach based on the use of barbed knotless bidirectional reabsorbable sutures named barbed reposition pharyngoplasty (BRP). All patients are submitted to bilateral tonsillectomy, and once PPM is demarcated, a partial incision is made at its inferior portion. A triangular-shaped mucosal and submucosal portion of the tonsillar fossa at its apex is removed in order to widen the oropharyngeal inlet. Then, the barbed sutured is utilized to superolaterally add tension to the upper portion of PPM and to stiffen LPW and the palate. In particular, the first pass of the needle is introduced at the center of the palate and passed laterally, reaching the pterygomandibular raphe. Passing around the pterygomandibular raphe, the needle reaches the upper portion of PPM through the tonsillar fossa. In this way, the muscular fibers are pulled up and anchored at the pterygomandibular raphe. The same procedure is applied to the other side. Effective and simple to perform, this technique has shown important results as both a single-stage procedure and a multilevel procedure associated with other upper airway corrective surgeries like base of tongue resection, hyoid suspension, and nasal surgery [26,27]. To minimize mucosal and muscular resection of the uvula, M.A. Babademez et al. [20] suggested some modifications using a monodirectional reabsorbable barbed thread to expand the upper part of the oropharyngeal inlet in a more conservative manner. In fact, in order to pull the uvula forward and superiorly, once PPM is fixed to the pterygomandibular raphe, the suture is passed horizontally through the root of the uvula to reach the opposite tonsillar bed, and then, PPM is anchored to the pterygomandibular raphe in the same fashion.

#### 4.2.3. Minimal Handling of Muscular Fibers

In order to further reduce the complications linked to muscle fiber section and fibrotic tissue development, some authors have proposed some minimally invasive and nonresective procedures. In 2015, Mantovani et al., modifying their previous “Roman blinds technique” for the treatment of retropalatal collapse [21,28], presented the barbed Roman blinds technique (BRBT), in which complete preservation of oropharyngeal fibromuscular structures is encouraged, and stiffening of LPW and of the soft palate is obtained through the use of a bidirectional barbed suture. With the aim of exposing the muscular fibers of PPM, a mucosectomy of the tonsillar fossa is performed. The first needle of the bidirectional barbed suture is inserted in the palatal mucosa 1 cm in front of the posterior nasal spine. The suture is passed inferiorly, following the periosteal layer, to reach the periuvular extremity of PPM. Once the muscular fibers are grasped, the suture is fixed to the pterygoid hamulus and passed in a craniocaudal direction, encircling the pterygomandibular raphe. The needle is then passed medially, reaching PPM to anchor it to the pterygomandibular raphe. Once the muscular fibers are fixed laterally, the suture is directed to the hamulus, and then, to the first insertion point. A nonresective technique, barbed suspension pharyngoplasty (BSP), was also presented in 2019 by M. Barbieri et al. [24]. After a tonsillar fossa mucosectomy or a bilateral tonsillectomy, as in BRBT, no further tissue dissection is executed, but a bidirectional barbed suture is performed in order to lateralize LPW and to give more tension to the soft palate. In particular, the needle is first inserted in the palatal mucosa at the level of the posterior nasal spine and passed anterolaterally towards the upper part of the tonsillar fossa. Then, once PPM is anchored by multiple stitches to the anterior pillar, the suture is passed laterally towards the pterygomandibular raphe and then it medially encircles the contralateral raphe and is directed back to the ipsilateral raphe, passing through the base of the uvula. In this way, a stronger tension is applied to the soft palate, and a more secure suture is achieved. As previously reported, the pterygomandibular raphe is considered an important security point by many authors, and some of them perform a tissue dissection in order to expose it, so as to better distribute the strongpoints during the suture. Hsueh-Yu Li et al. [23] proposed a suspension palatoplasty, in which, after a mucosal incision from the anterior pillar rim to 1 cm in front of the center mark of the pterygomandibular raphe, the submucosal tissue is dissected to expose the fibers of the raphe and a bilateral tonsillectomy is performed. The upper third of PPM is then fixed at the pterygomandibular raphe at different points in a craniocaudal direction, and the anterior and posterior pillars are sutured together. No major complications have been reported except for a transient globus sensation. Not all the procedures acting to stabilize LPW have considered the removal of palatal lymphoid tissue as a required step. In this way, for those patients with LPW collapse without tonsillar hypertrophy, M.A. El-Ahl and M.W. El-Anwar [22] proposed a modified expansion pharyngoplasty without tonsillectomy and any pharyngeal tissue ablation, in which a reabsorbable submucosal suture is performed in order to fix the upper portions of PPM and PGM to the pterygoid hamulus.

In all the papers included in the review, a statistically significant improvement of the apnea–hypopnea index (AHI) was reported, showing a considerable impact of LPW surgery in the restoration of efficient airflow. Some complications have been reported, but none of them were of a major nature. Nasal regurgitation, dysphagia, foreign body sensation, velopharyngeal insufficiency, taste loss, and sensation of oral dryness have been the most noted postoperative symptoms, which resolved spontaneously after few weeks of the surgery. In a few cases, postoperative bleeding was reported, and, when a barbed suture was used, partial suture extrusion was described, without any additional problems. To better evaluate the surgical contributions to the amelioration of LPW collapse, some limits of the examined papers need to be mentioned. The small number of samples and the retrospective modality of the study are the most significant restrictions encountered, allowing, in this way, a partial estimation of the results. In addition, in some papers, the LPW surgical technique was presented as part of a multiple-level treatment and not as a single procedure, partially hiding the real impact on clinical improvement provided by the stiffening of the pharyngeal structures. Further efforts need to be made in order to clarify the authentic role of LPW surgery, and in this way, the quantification of upper airway collapsibility represents an important tool in order to perform an attentive selection of the candidate and thorough monitoring of the clinical data. The pharyngeal critical closing pressure (Pcrit) is the most accurate index of upper airway collapsibility, but the invasivity and the complexity of its measurement do not make it clinically practical. With regard to this, A.M. Osman et al. [29] recently presented the peak inspiratory flow percentage (PIF%) as a marker of the collapsibility of the upper airways; it is related to Pcrit and easy to obtain during a routine CPAP titration study. PIF% could represent an important opportunity to better quantify the structural collapsibility of the upper airways in clinical practice, allowing more direct estimation of the effective value of LPW surgery and optimizing, in this way, the treatment of OSAS patients. 

Finally, the role of DISE as a phenotyping selection tool for surgical candidates also needs to be discussed. In fact, DISE appears to be a promising method for properly targeted therapy planning, in particular, allowing the selection or ruling-out of patients for specific surgical procedures [30].

## 5. Conclusions

Usually, upper airway collapse in patients with OSAS has a multilevel etiology, and LPW hypotonicity and flexibility represent two of the main factors. In this way, stiffening of the fibromuscular components and ablation of the redundant soft tissue of LPW constitute critical targets for many surgeons in order to restore correct airflow. Several surgical procedures acting on different fibromuscular structures have been proposed, each with different degrees of invasivity. With the improvements in surgical materials and knowledge acquisition around the physiopathology of LPW, surgeons have elaborated on increasingly efficient techniques to reduce the extension of tissue dissection and ablation. Many of them have reported important results, and while in some cases, postoperative complications have been experienced, these are usually temporary and of a small nature. Despite the high variability between the methods, all authors agree that meticulous preoperative analysis and selection of patients will reduce, as much as possible, the surgical failures. In this way, the absence of a gold standard, the possibility of utilizing the surgery of LPW in a multilevel context, the presence of a wide range of surgical options, and the short learning curve of many of them represent an opportunity to apply the most suitable method, according to the anatomical and clinical characteristics of the patient, to restore proper airflow.

## Figures and Tables

**Table 1:** Clinical data of papers included in the review.

Title	Structural Target and Modification of LPW	Number of Patients	Mean AHI Pre-Op	Mean AHI Post-Op	∆ AHI.	Follow-Up Time (Months)	Complications Described and Relating Incidence
Lateral Pharyngoplasty: A New Treatment for Obstructive Sleep Hypopnea Syndrome [7]	SPC	10	45.8	15.2	30.6	8.2	Oronasal reflux (10%), taste loss (10%).
Lateral-Expansion Pharyngoplasty: Combined Technique for the Treatment of Obstructive Sleep Apnea Syndrome [10]	SPC	38	22.4	13.6	8.8	7	-
Relocation Pharyngoplasty for Obstructive Sleep Apnea [11]	SPC	10	43.4	15.7	27.7	6	-
Anterolateral Advancement Pharyngoplasty: A New Technique for Treatment of Obstructive Sleep Apnea [12]	SPC	38	42.1	16.3	25.8	6	-
Expansion Sphincter Pharyngoplasty: A New Technique for the Treatment of Obstructive Sleep Apnea [13]	PPM section	45	44.2	12.0	32.2	6.5	-
Functional Expansion Pharyngoplasty in the Treatment of Obstructive Sleep Apnea [14]	PPM section	85	33.3	11.7	21.6	6	Postsurgical bleeding (2.3%).
Soft Palatal Webbing Flap Palatopharyngoplasty for Both Soft Palatal and Obstructive Sleep Apnea: A New Innovative Technique without Tonsillectomy [15]	PPM section	28	46.1	11	35.1	6	Excessive postnasal discharge (10.7%) temporary velopharyngeal insufficiency (7.1%), sensation of oral dryness (25%).
Modified Barbed Soft Palatal Posterior Pillar Webbing Flap Palatopharyngoplasty [16]	PPM section	21	47.7	12.3	35.4	6	Temporary velopharyngeal insufficiency (5%), excessive postnasal discharge (19%).
Modified Expansion Sphincter pharyngoplasty for Treatment of Children with Obstructive Sleep Apnea [17]	PPM partial section	25	60.5	2.0	58.5	-	Postoperative bleeding (4%).
A Modified Uvulopalatal Flap with Lateral Pharyngoplasty for Treatment in 92 Adults with Obstructive Sleep Apnoea Syndrome [18]	PPM partial section	92	39.1	7.9	21,2	6	Nasal regurgitation, bleeding, dysphagia, Foreign body sensation.
Barbed Reposition Pharyngoplasty (BRP) for OSAHS: A Feasibility, Safety, Efficacy and Teachability Pilot Study. “We are on the giant’s shoulders” [19]	PPM partial section	10	43.65	13.57	30.08	6	Foreign body sensation, partial thread extrusion (20%).
Technical Update of Barbed Pharyngoplasty for Retropalatal Obstruction in Obstructive Sleep Apnoea [20]	PPM partial section	17	29.9	5.4	24.5	6	Foreign body sensation.
Barbed Roman Blinds Technique for Treatment of Obstructive Sleep Apnea: How We Do It? [21]	PPM minimal handling	32	36.9	13.7	23.2	12	-
Expansion Pharyngoplasty by New Simple Suspension Sutures without Tonsillectomy [22]	PPM minimal handling	24	28.6	8.9	19.7	9	-
Suspension Palatoplasty for Obstructive Sleep Apnea- Preliminary Study [23]	PPM minimal handling	25	39.8	15.1	24.7	6	Globus sensation of the throat.
Barbed Suspension Pharyngoplasty for Treatment of Lateral Pharyngeal Wall and Palatal Collapse in Patients Affected by OSAHS [24]	PPM minimal handling	20	25	5	20	6	Transient velopharyngeal insufficiency (10%), Partial thread extrusion (25%).
